# Sharing Detailed Research Data Is Associated with Increased Citation Rate

**DOI:** 10.1371/journal.pone.0000308

**Published:** 2007-03-21

**Authors:** Heather A. Piwowar, Roger S. Day, Douglas B. Fridsma

**Affiliations:** Department of Biomedical Informatics, University of Pittsburgh School of Medicine, Pittsburgh, Pennsylvania, United States of America; University of Ioannina School of Medicine, Greece

## Abstract

**Background:**

Sharing research data provides benefit to the general scientific community, but the benefit is less obvious for the investigator who makes his or her data available.

**Principal Findings:**

We examined the citation history of 85 cancer microarray clinical trial publications with respect to the availability of their data. The 48% of trials with publicly available microarray data received 85% of the aggregate citations. Publicly available data was significantly (p = 0.006) associated with a 69% increase in citations, independently of journal impact factor, date of publication, and author country of origin using linear regression.

**Significance:**

This correlation between publicly available data and increased literature impact may further motivate investigators to share their detailed research data.

## Introduction

Sharing information facilitates science. Publicly sharing detailed research data–sample attributes, clinical factors, patient outcomes, DNA sequences, raw mRNA microarray measurements–with other researchers allows these valuable resources to contribute far beyond their original analysis[1]. In addition to being used to confirm original results, raw data can be used to explore related or new hypotheses, particularly when combined with other publicly available data sets. Real data is indispensable when investigating and developing study methods, analysis techniques, and software implementations. The larger scientific community also benefits: sharing data encourages multiple perspectives, helps to identify errors, discourages fraud, is useful for training new researchers, and increases efficient use of funding and patient population resources by avoiding duplicate data collection.

Believing that that these benefits outweigh the costs of sharing research data, many initiatives actively encourage investigators to make their data available. Some journals, including the *PLoS* family, require the submission of detailed biomedical data to publicly available databases as a condition of publication[2]–[4]. Since 2003, the NIH has required a data sharing plan for all large funding grants. The growing open-access publishing movement will perhaps increase peer pressure to share data.

However, while the general research community benefits from shared data, much of the burden for sharing the data falls to the study investigator. Are there benefits for the investigators themselves?

A currency of value to many investigators is the number of times their publications are cited. Although limited as a proxy for the scientific contribution of a paper[5], citation counts are often used in research funding and promotion decisions and have even been assigned a salary-increase dollar value[6]. Boosting citation rate is thus is a potentially important motivator for publication authors.

In this study, we explored the relationship between the citation rate of a publication and whether its data was made publicly available. Using cancer microarray clinical trials, we addressed the following questions: Do trials which share their microarray data receive more citations? Is this true even within lower profile trials? What other data-sharing variables are associated with an increased citation rate? While this study is not able to investigate causation, quantifying associations is a valuable first step in understanding these relationships. Clinical microarray data provides a useful environment for the investigation: despite being valuable for reuse and extremely costly to collect, is not yet universally shared.

## Results

We studied the citations of 85 cancer microarray clinical trials published between January 1999 and April 2003, as identified in a systematic review by Ntzani and Ioannidis[7] and listed in Supplementary Text S1. We found 41 of the 85 clinical trials (48%) made their microarray data publicly available on the internet. Most data sets were located on lab websites (28), with a few found on publisher websites (4), or within public databases (6 in the Stanford Microarray Database (SMD)[8], 6 in Gene Expression Omnibus (GEO)[9], 2 in ArrayExpress[10], 2 in the NCI GeneExpression Data Portal (GEDP)(gedp.nci.nih.gov); some datasets in more than one location). The internet locations of the datasets are listed in Supplementary Text S2. The majority of datasets were made available concurrently with the trial publication, as illustrated within the WayBackMachine internet archives (www.archive.org/web/web.php) for 25 of the datasets and mention of supplementary data within the trial publication itself for 10 of the remaining 16 datasets. As seen in Table 1, trials published in high impact journals, prior to 2001, or with US authors were more likely to share their data.

**Table 1:** Characteristics of Eligible Trials by Data Sharing.

	Number of Articles			Odds Ratio (95% confidence interval)
	Total	Data Shared	Data Not Shared	
**TOTAL**	**85**	**41 (48%)**	**44 (52%)**	
**High Impact (> = 25)**	12	12 (100%)	0 (0%)	∞ (3.8 to ∞)
**Low Impact Journal**	73	29 (40%)	44 (60%)	
**Published 1999–2000**	6	5 (83%)	1 (17%)	6.0 (0.6 to 288.5)
**Published 2001–2003**	79	36 (46%)	43 (54%)	
**Include a US Author**	56	35 (63%)	21 (38%)	6.4 (2.0 to 21.9)
**No US Authors**	29	6 (21%)	23 (79%)	

**Figure 1:**
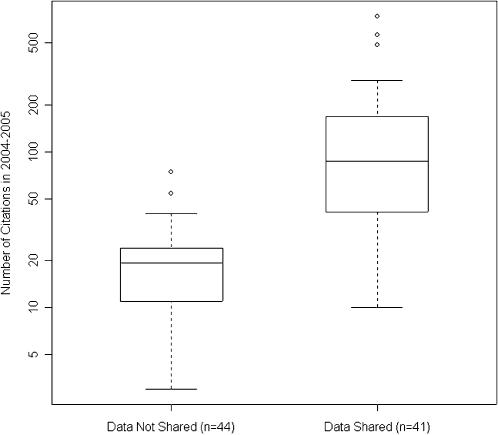
Distribution of 2004–2005 citation counts of 85 trials by data availability. The 41 clinical trial publications which publicly shared their microarray data received more citations, in general, than the 44 publications which did not share their microarray data. In this plot of the distribution of citation counts received by each publication, the extent of the box encompasses the interquartile range of the citation counts, whiskers extend to 1.5 times the interquartile range, and lines within the boxes represent medians.

The cohort of 85 trials was cited an aggregate of 6239 times in 2004–2005 by 3133 distinct articles (median of 1.0 cohort citation per article, range 1–23). The 48% of trials which shared their data received a total of 5334 citations (85% of aggregate), distributed as shown in Figure 1.

Whether a trial's dataset was made publicly available was significantly associated with the log of its 2004–2005 citation rate (69% increase in citation count; 95% confidence interval: 18 to 143%, p = 0.006), independent of journal impact factor, date of publication, and US authorship. Detailed results of this multivariate linear regression are given in Table 2. A similar result was found when we regressed on the number of citations each trial received during the 24 months after its publication (45% increase in citation count; 95% confidence interval: 1 to 109%, p = 0.050).

**Table 2:** Multivariate regression on citation count for 85 publications

	Percent increase in citation count (95% confidence interval)	p-value
Publish in a journal with twice the impact factor	84% (59 to 109%)	<0.001
Increase the publication date by a month	−3% (−5 to −2%)	<0.001
Include a US author	38% (1 to 89%)	0.049
**Make data publicly available**	**69% (18 to 143%)**	**0.006**

We calculated a multivariate linear regression over the citation counts, including covariates for journal impact factor, date of publication, US authorship, and data availability. The coefficients and p-values for each of the covariates are shown here, representing the contribution of each covariate to the citation count, independent of other covariates.

**Figure 2:**
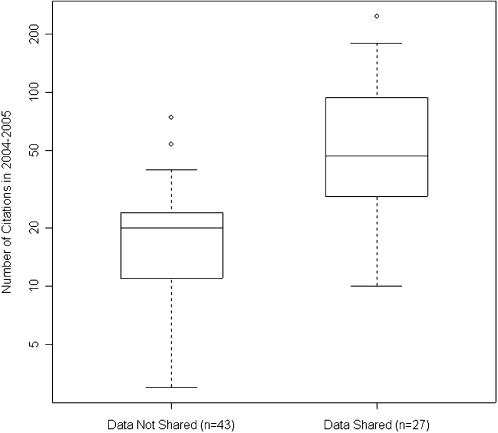
Distribution of 2004–2005 citation counts of the 70 lower-profile trials by data availability. For trials which were published after 2000 and in journals with an impact factor less than 25, the 27 clinical trial publications which publicly shared their microarray data received more citations, in general, than the 43 publications which did not share their microarray data. In this plot of the distribution of citation counts received by each publication, the extent of the box encompasses the interquartile range of the citation counts, whiskers extend to 1.5 times the interquartile range, and lines within the boxes represent medians.

To confirm that these findings were not dependent on a few extremely high-profile papers, we repeated our analysis on a subset of the cohort. We define papers published after the year 2000 in journals with an impact factor less than 25 as lower-profile publications. Of the 70 trials in this subset, only 27 (39%) made their data available, although they received 1875 of 2761 (68%) aggregate citations. The distribution of the citations by data availability in this subset is shown in Figure 2. The association between data sharing and citation rate remained significant in this lower-profile subset, independent of other covariates within a multivariate linear regression (71% increase in citation count; 95% confidence interval: 19 to 146%, p = 0.005).

Lastly, we performed exploratory analysis on citation rate within the subset of trials which shared their microarray data; results are given in Table 3 and raw covariate data in Supplementary Data S1. The number of patients in a trial and a clinical endpoint correlated with increased citation rate. Assuming shared data is actually re-analyzed, one might expect an increase in citations for those trials which generated data on a standard platform (Affymetrix), or released it in a central location or format (SMD, GEO, GEDP)[11]. However, the choice of platform was insignificant and only those trials located in SMD showed a weak trend of increased citations. In fact, the 6 trials with data in GEO (in addition to other locations for 4 of the 6) actually showed an inverse relationship to citation rate, though we hesitate to read much into this due to the small number of trials in this set. The few trials in this cohort which, in addition to gene expression fold-change or other preprocessed information, shared their raw probe data or actual microarray images did not receive additional citations. Finally, although finding diverse microarray datasets online is non-trivial, an additional increase in citations was not noted for trials which mentioned their Supplementary Material within their paper, nor for those trials with datasets identified by a centralized, established data mining website. In summary, only trial design features such as size and clinical endpoint showed a significant association with citation rate; covariates relating to the data collection and how the data was made available only showed very weak trends. Perhaps with a larger and more balanced sample of trials with shared data these trends would be more clear.

**Table 3:** Exploratory regressions on citation count for the 41 publications with shared data

	Number of articles (% of total)	Number of citations (% of total)	Percent increase in citation count	p-value
**TOTAL**	**41**	**5334**		
Trial size>25 patients	26 (63%)	3704 (69%)	122%	<0.001
Clinical endpoint	18 (44%)	3404 (64%)	79%	0.01
Affymetrix platform	22 (54%)	2735 (51%)	18%	0.43
In GEO database	6 (15%)	939 (18%)	−52%	0.02
In SMD database	6 (15%)	1114 (21%)	24%	0.48
Raw data available	20 (49%)	2437 (46%)	−2%	0.91
Pub mentions Suppl. Data	35 (85%)	4854 (91%)	11%	0.73
Has Oncomine profile	35 (85%)	4884 (92%)	19%	0.54

The coefficient and p-value for each covariate in the table were calculated from separate multivariate linear regressions over the citation count, including covariates for journal impact factor, date of publication, and US authorship.

## Discussion

We found that cancer clinical trials which share their microarray data were cited about 70% more frequently than clinical trials which do not. This result held even for lower-profile publications and thus is relevant to authors of all trials.

A parallel can be drawn between making study data publicly available and publishing a paper itself in an open-access journal. The association with an increased citation rate is similar[12]. While altruism no doubt plays a part in the motivation of authors in both cases, studies have found that an additional reason authors choose to publish in open-access journals is that they believe their articles will be cited more frequently[13], [14], endorsing the relevance of our result as a potential motivator.

We note an important limitation of this study: the demonstrated association does not imply causation. Receiving many citations and sharing data may stem from a common cause rather than being directly causally related. For example, a large, high-quality, clinically important trial would naturally receive many citations due to its medical relevance; meanwhile, its investigators may be more inclined to share its data than they would be for a smaller trial-perhaps due greater resources or confidence in the results.

Nonetheless, if we speculate for a moment that some or all of the association is indeed causal, we can hypothesize several mechanisms by which making data available may increase citations. The simplest mechanism is due to increased exposure: listing the dataset in databases and on websites will increase the number of people who encounter the publication. These people may then subsequently cite it for any of the usual reasons one cites a paper, such as paying homage, providing background reading, or noting corroborating or disputing claims ([15] provides a summary of research into citation behavior). More interestingly, evidence suggests that shared microarray data is indeed often reanalyzed[16], so at least some of the additional citations are certainly in this context. Finally, these re-analyses may spur enthusiasm and synergy around a specific research question, indirectly focusing publications and increasing the citation rate of all participants. These hypotheses are not tested in this study: additional research is needed to study the context of these citations and the degree, variety, and impact of any data re-use. Further, it would be interesting to assess the impact of reuse on the community, quantifying whether it does in fact lead to collaboration, a reduction in resource use, and scientific advances.

Since it is generally agreed that sharing data is of value to the scientific community[16]–[21], it is disappointing that less than half of the trials we looked at made their data publicly available. It is possible that attitudes may have changed in the years since these trials were published, however even recent evidence (in a field tangential to microarray trials) demonstrates a lack of willingness and ability to share data: an analysis in 2005 by Kyzas *et al*.[22] found that primary investigators for 17 of 63 studies on TP53 status in head and neck squamous cell carcinoma did not respond to a request for additional information, while 5 investigators replied they were unable to retrieve raw data.

Indeed, there are many personal difficulties for those who undertake to share their data[1]. A major cost is time: the data have to be formatted, documented, and released. Unfortunately this investment is often larger than one might guess: in the realm of microarray and particularly clinical information, it is nontrivial to decide what data to release, how to de-identify it, how to format it, and how to document it. Further, it is sometimes complicated to decide where to best publish data, since supplementary information and laboratory sites are transient[23], [24] Beyond a time investment, releasing data can induce fear. There is a possibility that the original conclusions may be challenged by a re-analysis, whether due to possible errors in the original study[25], a misunderstanding or misinterpretation of the data[26], or simply more refined analysis methods. Future data miners might discover additional relationships in the data, some of which could disrupt the planned research agenda of the original investigators. Investigators may fear they will be deluged with requests for assistance, or need to spend time reviewing and possibly rebutting future re-analyses. They might feel that sharing data decreases their own competitive advantage, whether future publishing opportunities, information trade-in-kind offers with other labs, or potentially profit-making intellectual property. Finally, it can be complicated to release data. If not well-managed, data can become disorganized and lost. Some informed consent agreements may not obviously cover subsequent uses of data. De-identification can be complex. Study sponsors, particularly from industry, may not agree to release raw detailed information. Data sources may be copyrighted such that the data subsets can not be freely shared, though it is always worth asking.

Although several of these difficulties are challenging to overcome, many are being addressed by a variety of initiatives, thereby decreasing the barriers to data sharing. For example, within the area of microarray clinical trials, several public microarray databases (SMD[27], GEO[9], ArrayExpress[10], CIBEX[28], GEDP(gedp.nci.nih.gov)) offer an obvious, centralized, free, and permanent data storage solution. Standards have been developed to specify minimal required data elements (MIAME[29] for microarray data, REMARK[30] for prognostic study details), consistent data encoding (MAGE-ML[31] for microarray data), and semantic models (BRIDG (www.bridgproject.org) for study protocol details). Software exists to help de-identify some types of patient records (De-ID[32]). The NIH and other agencies allow funds for data archiving and sharing. Finally, large initiatives (NCI's caBIG[33]) are underway to build tools and communities to enable and advance sharing data.

Research consumes considerable resources from the public trust. As data sharing gets easier and benefits are demonstrated for the individual investigator, hopefully authors will become more apt to share their study data and thus maximize its usefulness to society.

In the spirit of this analysis, we have made publicly available the bibliometric detailed research data compiled for this study (see Supplementary Information and http://www.pitt.edu/∼hap7).

## Materials and Methods

### Identification and Eligibility of Relevant Studies

We compared the citation impact of clinical trials which made their cancer microarray data publicly available to the citation impact of trials which did not. A systematic review by Ntzani and Ioannidis[7] identified clinical trials published between January 1999 and April 2003 which investigated correlations between microarray gene expression and human cancer outcomes and correlates. We adopted this set of 85 trials as the cohort of interest.

### Data Extraction

We assessed whether each of these trials made its microarray data publicly available by examining a variety of publication and internet resources. Specifically, we looked for mention of Supplementary Information within the trial publication, searched the Stanford Microarray Database (SMD)[8], Gene Expression Omnibus (GEO)[9], ArrayExpress[10], CIBEX[28], and the NCI GeneExpression Data Portal (GEDP)(gedp.nci.nih.gov), investigated whether a data link was provided within Oncomine[34], and consulted the bibliography of data re-analyses. Microarray data release was not required by any journals within the timeframe of these trial publications. Some studies may make their data available upon individual request, but this adds a burden to the data user and so was not considered “publicly available” for the purposes of this study.

We attempted to determine the date data was made available through notations in the published paper itself and records within the WayBackMachine internet archive (www.archive.org/web/web.php). Inclusion in the WayBackMachine archive for a given date proves a resource was available, however, because archiving is not comprehensive, absence from the archive does not itself demonstrate a resource did not exist on that date.

The citation history for each trial was collected through the Thomson Scientific Institute for Scientific Information (ISI) Science Citation Index at the Web of Science Database (www.isinet.com). Only citations with a document type of ‘Article’ were considered, thus excluding citations by reviews, editorials, and other non-primary research papers.

For each trial, we also extracted the impact factor of the publishing journal (ISI Journal Citation Reports 2004), the date of publication, and the address of the authors from the ISI Web of Science. Trial size, clinical endpoint, and microarray platform were extracted from the Ntzani and Ioannidis review[7].

### Analysis

The main analyses addressed the number of citations each trial received between January 2004 and December 2005. Because the pattern of citations rates is complex–changing not only with duration since publication but also with maturation of the general microarray field–a confirmatory analysis was performed using the number of citations each publication received within the first 24 months of its publication.

Although citation patterns covering a broad scope of literature types are left-skewed[35], we verified that citation rates within our relatively homogeneous cohort were roughly log-normal and thus used parametric statistics.

Multivariate linear regression was used to evaluate the association between the public availability of a trial's microarray data and number of citations (after log transformation) it received. The impact factor of the journal which published each trial, the date of publication, and the country of authors are known to correlate to citation rate[36], so these factors were included as covariates. Impact factor was log-transformed, date of publication was measured as months since January 1999, and author country was coded as 1 if any investigator has a US address and 0 otherwise.

Since seminal papers–often those published early in the history a field or in very high-impact journals–receive an unusually high number of citations, we performed a subset analysis to determine whether our results held when considering only those trials which were published after 2000 and in lower-impact (<25) journals.

Finally, as exploratory analysis within the subset of all trials with publicly available microarray data, we looked at the linear regression relationships between additional covariates and citation count. Covariates included trial size, clinical endpoint, microarray platform, inclusion in various public databases, release of raw data, mention of supplementary information, and reference within the Oncomine[34] repository.

Statistical analysis was performed using the stats package in R version 2.1[37]; the code is included as Supplementary Text S3. P-values are two-tailed.

## Supporting Information

Text S1 Cohort Publication Bibliography
(0.05 MB DOC)

Text S2 Locations of Publicly Available Data for the Cohort
(0.05 MB DOC)

Text S3 Statistical Analysis R-code
(0.01 MB TXT)

Data S1 Raw Citation Counts and Covariates
(0.04 MB XLS)
