# Supplementary material for: Sharing Detailed Research Data Is Associated with Increased Citation Rate
Source: PLoS One. 2007 Mar 21;2(3):e308. doi: 10.1371/journal.pone.0000308 (PMC1817752; doi:10.1371/journal.pone.0000308)
Supplement: Text S1 — Cohort Publication Bibliography (0.05 MB DOC) [file pone.0000308.s001.doc]

#### Sharing Detailed Research Data is

#### Associated with Increased Citation Rate

**The Cohort of Trial Publications**

**The 85 cancer microarray trials published before early 2003 identified in a systematic review by Ntzani and Ioannidis**

Ntzani EE, Ioannidis JP (2003) Predictive ability of DNA microarrays for cancer outcomes and correlates: an empirical assessment. Lancet 362: 1439-1444

1. Adeyinka A, Emberley E, Niu Y, Snell L, Murphy LC, et al. (2002) Analysis of gene expression in ductal carcinoma in situ of the breast. Clin Cancer Res 8: 3788-3795.

2. Ahr A, Karn T, Solbach C, Seiter T, Strebhardt K, et al. (2002) Identification of high risk breast-cancer patients by gene expression profiling. Lancet 359: 131-132.

3. Alizadeh AA, Eisen MB, Davis RE, Ma C, Lossos IS, et al. (2000) Distinct types of diffuse large B-cell lymphoma identified by gene expression profiling. Nature 403: 503-511.

4. Bayani J, Brenton JD, Macgregor PF, Beheshti B, Albert M, et al. (2002) Parallel analysis of sporadic primary ovarian carcinomas by spectral karyotyping, comparative genomic hybridization, and expression microarrays. Cancer Res 62: 3466-3476.

5. Beer DG, Kardia SL, Huang CC, Giordano TJ, Levin AM, et al. (2002) Gene-expression profiles predict survival of patients with lung adenocarcinoma. Nat Med 8: 816-824.

6. Belbin TJ, Singh B, Barber I, Socci N, Wenig B, et al. (2002) Molecular classification of head and neck squamous cell carcinoma using cDNA microarrays. Cancer Res 62: 1184-1190.

7. Bertucci F, Nasser V, Granjeaud S, Eisinger F, Adelaide J, et al. (2002) Gene expression profiles of poor-prognosis primary breast cancer correlate with survival. Hum Mol Genet 11: 863-872.

8. Bhattacharjee A, Richards WG, Staunton J, Li C, Monti S, et al. (2001) Classification of human lung carcinomas by mRNA expression profiling reveals distinct adenocarcinoma subclasses. Proc Natl Acad Sci U S A 98: 13790-13795.

9. Birkenkamp-Demtroder K, Christensen LL, Olesen SH, Frederiksen CM, Laiho P, et al. (2002) Gene expression in colorectal cancer. Cancer Res 62: 4352-4363.

10. Bittner M, Meltzer P, Chen Y, Jiang Y, Seftor E, et al. (2000) Molecular classification of cutaneous malignant melanoma by gene expression profiling. Nature 406: 536-540.

11. Bohen SP, Troyanskaya OG, Alter O, Warnke R, Botstein D, et al. (2003) Variation in gene expression patterns in follicular lymphoma and the response to rituximab. Proc Natl Acad Sci U S A 100: 1926-1930.

12. Cohen N, Rozenfeld-Granot G, Hardan I, Brok-Simoni F, Amariglio N, et al. (2001) Subgroup of patients with Philadelphia-positive chronic myelogenous leukemia characterized by a deletion of 9q proximal to ABL gene: expression profiling, resistance to interferon therapy, and poor prognosis. Cancer Genet Cytogenet 128: 114-119.

13. Delpuech O, Trabut JB, Carnot F, Feuillard J, Brechot C, et al. (2002) Identification, using cDNA macroarray analysis, of distinct gene expression profiles associated with pathological and virological features of hepatocellular carcinoma. Oncogene 21: 2926-2937.

14. Devilard E, Bertucci F, Trempat P, Bouabdallah R, Loriod B, et al. (2002) Gene expression profiling defines molecular subtypes of classical Hodgkin's disease. Oncogene 21: 3095-3102.

15. Dhanasekaran SM, Barrette TR, Ghosh D, Shah R, Varambally S, et al. (2001) Delineation of prognostic biomarkers in prostate cancer. Nature 412: 822-826.

16. Dyrskjot L, Thykjaer T, Kruhoffer M, Jensen JL, Marcussen N, et al. (2003) Identifying distinct classes of bladder carcinoma using microarrays. Nat Genet 33: 90-96.

17. El-Naggar AK, Kim HW, Clayman GL, Coombes MM, Le B, et al. (2002) Differential expression profiling of head and neck squamous carcinoma: significance in their phenotypic and biological classification. Oncogene 21: 8206-8219.

18. Fathallah-Shaykh HM, Rigen M, Zhao LJ, Bansal K, He B, et al. (2002) Mathematical modeling of noise and discovery of genetic expression classes in gliomas. Oncogene 21: 7164-7174.

19. Fritz B, Schubert F, Wrobel G, Schwaenen C, Wessendorf S, et al. (2002) Microarray-based copy number and expression profiling in dedifferentiated and pleomorphic liposarcoma. Cancer Res 62: 2993-2998.

20. Fuller GN, Hess KR, Rhee CH, Yung WK, Sawaya RA, et al. (2002) Molecular classification of human diffuse gliomas by multidimensional scaling analysis of gene expression profiles parallels morphology-based classification, correlates with survival, and reveals clinically-relevant novel glioma subsets. Brain Pathol 12: 108-116.

21. Fuller GN, Rhee CH, Hess KR, Caskey LS, Wang R, et al. (1999) Reactivation of insulin-like growth factor binding protein 2 expression in glioblastoma multiforme: a revelation by parallel gene expression profiling. Cancer Res 59: 4228-4232.

22. Garber ME, Troyanskaya OG, Schluens K, Petersen S, Thaesler Z, et al. (2001) Diversity of gene expression in adenocarcinoma of the lung. Proc Natl Acad Sci U S A 98: 13784-13789.

23. Golub TR, Slonim DK, Tamayo P, Huard C, Gaasenbeek M, et al. (1999) Molecular classification of cancer: class discovery and class prediction by gene expression monitoring. Science 286: 531-537.

24. Gruvberger S, Ringner M, Chen Y, Panavally S, Saal LH, et al. (2001) Estrogen receptor status in breast cancer is associated with remarkably distinct gene expression patterns. Cancer Res 61: 5979-5984.

25. Gutmann DH, Hedrick NM, Li J, Nagarajan R, Perry A, et al. (2002) Comparative gene expression profile analysis of neurofibromatosis 1-associated and sporadic pilocytic astrocytomas. Cancer Res 62: 2085-2091.

26. Hedenfalk I, Duggan D, Chen Y, Radmacher M, Bittner M, et al. (2001) Gene-expression profiles in hereditary breast cancer. N Engl J Med 344: 539-548.

27. Hippo Y, Taniguchi H, Tsutsumi S, Machida N, Chong JM, et al. (2002) Global gene expression analysis of gastric cancer by oligonucleotide microarrays. Cancer Res 62: 233-240.

28. Hofmann WK, de Vos S, Elashoff D, Gschaidmeier H, Hoelzer D, et al. (2002) Relation between resistance of Philadelphia-chromosome-positive acute lymphoblastic leukaemia to the tyrosine kinase inhibitor STI571 and gene-expression profiles: a gene-expression study. Lancet 359: 481-486.

29. Huang H, Colella S, Kurrer M, Yonekawa Y, Kleihues P, et al. (2000) Gene expression profiling of low-grade diffuse astrocytomas by cDNA arrays. Cancer Res 60: 6868-6874.

30. Iizuka N, Oka M, Yamada-Okabe H, Mori N, Tamesa T, et al. (2002) Comparison of gene expression profiles between hepatitis B virus- and hepatitis C virus-infected hepatocellular carcinoma by oligonucleotide microarray data on the basis of a supervised learning method. Cancer Res 62: 3939-3944.

31. Iizuka N, Oka M, Yamada-Okabe H, Nishida M, Maeda Y, et al. (2003) Oligonucleotide microarray for prediction of early intrahepatic recurrence of hepatocellular carcinoma after curative resection. Lancet 361: 923-929.

32. Jazaeri AA, Lu K, Schmandt R, Harris CP, Rao PH, et al. (2003) Molecular determinants of tumor differentiation in papillary serous ovarian carcinoma. Mol Carcinog 36: 53-59.

33. Jazaeri AA, Yee CJ, Sotiriou C, Brantley KR, Boyd J, et al. (2002) Gene expression profiles of BRCA1-linked, BRCA2-linked, and sporadic ovarian cancers. J Natl Cancer Inst 94: 990-1000.

34. Kihara C, Tsunoda T, Tanaka T, Yamana H, Furukawa Y, et al. (2001) Prediction of sensitivity of esophageal tumors to adjuvant chemotherapy by cDNA microarray analysis of gene-expression profiles. Cancer Res 61: 6474-6479.

35. Klein U, Tu Y, Stolovitzky GA, Mattioli M, Cattoretti G, et al. (2001) Gene expression profiling of B cell chronic lymphocytic leukemia reveals a homogeneous phenotype related to memory B cells. J Exp Med 194: 1625-1638.

36. LaTulippe E, Satagopan J, Smith A, Scher H, Scardino P, et al. (2002) Comprehensive gene expression analysis of prostate cancer reveals distinct transcriptional programs associated with metastatic disease. Cancer Res 62: 4499-4506.

37. Lee S, Baek M, Yang H, Bang YJ, Kim WH, et al. (2002) Identification of genes differentially expressed between gastric cancers and normal gastric mucosa with cDNA microarrays. Cancer Lett 184: 197-206.

38. Luo JH, Yu YP, Cieply K, Lin F, Deflavia P, et al. (2002) Gene expression analysis of prostate cancers. Mol Carcinog 33: 25-35.

39. MacDonald TJ, Brown KM, LaFleur B, Peterson K, Lawlor C, et al. (2001) Expression profiling of medulloblastoma: PDGFRA and the RAS/MAPK pathway as therapeutic targets for metastatic disease. Nat Genet 29: 143-152.

40. Magee JA, Araki T, Patil S, Ehrig T, True L, et al. (2001) Expression profiling reveals hepsin overexpression in prostate cancer. Cancer Res 61: 5692-5696.

41. Mendez E, Cheng C, Farwell DG, Ricks S, Agoff SN, et al. (2002) Transcriptional expression profiles of oral squamous cell carcinomas. Cancer 95: 1482-1494.

42. Miura K, Bowman ED, Simon R, Peng AC, Robles AI, et al. (2002) Laser capture microdissection and microarray expression analysis of lung adenocarcinoma reveals tobacco smoking- and prognosis-related molecular profiles. Cancer Res 62: 3244-3250.

43. Moos PJ, Raetz EA, Carlson MA, Szabo A, Smith FE, et al. (2002) Identification of gene expression profiles that segregate patients with childhood leukemia. Clin Cancer Res 8: 3118-3130.

44. Moran CJ, Arenberg DA, Huang CC, Giordano TJ, Thomas DG, et al. (2002) RANTES expression is a predictor of survival in stage I lung adenocarcinoma. Clin Cancer Res 8: 3803-3812.

45. Mukasa A, Ueki K, Matsumoto S, Tsutsumi S, Nishikawa R, et al. (2002) Distinction in gene expression profiles of oligodendrogliomas with and without allelic loss of 1p. Oncogene 21: 3961-3968.

46. Notterman DA, Alon U, Sierk AJ, Levine AJ (2001) Transcriptional gene expression profiles of colorectal adenoma, adenocarcinoma, and normal tissue examined by oligonucleotide arrays. Cancer Res 61: 3124-3130.

47. Ohmine K, Ota J, Ueda M, Ueno S, Yoshida K, et al. (2001) Characterization of stage progression in chronic myeloid leukemia by DNA microarray with purified hematopoietic stem cells. Oncogene 20: 8249-8257.

48. Okabe H, Satoh S, Kato T, Kitahara O, Yanagawa R, et al. (2001) Genome-wide analysis of gene expression in human hepatocellular carcinomas using cDNA microarray: identification of genes involved in viral carcinogenesis and tumor progression. Cancer Res 61: 2129-2137.

49. Perou CM, Jeffrey SS, van de Rijn M, Rees CA, Eisen MB, et al. (1999) Distinctive gene expression patterns in human mammary epithelial cells and breast cancers. Proc Natl Acad Sci U S A 96: 9212-9217.

50. Pomeroy SL, Tamayo P, Gaasenbeek M, Sturla LM, Angelo M, et al. (2002) Prediction of central nervous system embryonal tumour outcome based on gene expression. Nature 415: 436-442.

51. Rickman DS, Bobek MP, Misek DE, Kuick R, Blaivas M, et al. (2001) Distinctive molecular profiles of high-grade and low-grade gliomas based on oligonucleotide microarray analysis. Cancer Res 61: 6885-6891.

52. Rodriguez S, Jafer O, Goker H, Summersgill BM, Zafarana G, et al. (2003) Expression profile of genes from 12p in testicular germ cell tumors of adolescents and adults associated with i(12p) and amplification at 12p11.2-p12.1. Oncogene 22: 1880-1891.

53. Rosenwald A, Alizadeh AA, Widhopf G, Simon R, Davis RE, et al. (2001) Relation of gene expression phenotype to immunoglobulin mutation genotype in B cell chronic lymphocytic leukemia. J Exp Med 194: 1639-1647.

54. Rosenwald A, Wright G, Chan WC, Connors JM, Campo E, et al. (2002) The use of molecular profiling to predict survival after chemotherapy for diffuse large-B-cell lymphoma. N Engl J Med 346: 1937-1947.

55. Schoch C, Kohlmann A, Schnittger S, Brors B, Dugas M, et al. (2002) Acute myeloid leukemias with reciprocal rearrangements can be distinguished by specific gene expression profiles. Proc Natl Acad Sci U S A 99: 10008-10013.

56. Shipp MA, Ross KN, Tamayo P, Weng AP, Kutok JL, et al. (2002) Diffuse large B-cell lymphoma outcome prediction by gene-expression profiling and supervised machine learning. Nat Med 8: 68-74.

57. Shirota Y, Kaneko S, Honda M, Kawai HF, Kobayashi K (2001) Identification of differentially expressed genes in hepatocellular carcinoma with cDNA microarrays. Hepatology 33: 832-840.

58. Shridhar V, Lee J, Pandita A, Iturria S, Avula R, et al. (2001) Genetic analysis of early- versus late-stage ovarian tumors. Cancer Res 61: 5895-5904.

59. Signoretti S, Di Marcotullio L, Richardson A, Ramaswamy S, Isaac B, et al. (2002) Oncogenic role of the ubiquitin ligase subunit Skp2 in human breast cancer. J Clin Invest 110: 633-641.

60. Singh D, Febbo PG, Ross K, Jackson DG, Manola J, et al. (2002) Gene expression correlates of clinical prostate cancer behavior. Cancer Cell 1: 203-209.

61. Skotheim RI, Monni O, Mousses S, Fossa SD, Kallioniemi OP, et al. (2002) New insights into testicular germ cell tumorigenesis from gene expression profiling. Cancer Res 62: 2359-2364.

62. Smith MW, Yue ZN, Geiss GK, Sadovnikova NY, Carter VS, et al. (2003) Identification of novel tumor markers in hepatitis C virus-associated hepatocellular carcinoma. Cancer Res 63: 859-864.

63. Sorlie T, Perou CM, Tibshirani R, Aas T, Geisler S, et al. (2001) Gene expression patterns of breast carcinomas distinguish tumor subclasses with clinical implications. Proc Natl Acad Sci U S A 98: 10869-10874.

64. Sotiriou C, Powles TJ, Dowsett M, Jazaeri AA, Feldman AL, et al. (2002) Gene expression profiles derived from fine needle aspiration correlate with response to systemic chemotherapy in breast cancer. Breast Cancer Res 4: R3.

65. Stratowa C, Loffler G, Lichter P, Stilgenbauer S, Haberl P, et al. (2001) CDNA microarray gene expression analysis of B-cell chronic lymphocytic leukemia proposes potential new prognostic markers involved in lymphocyte trafficking. Int J Cancer 91: 474-480.

66. Takahashi M, Rhodes DR, Furge KA, Kanayama H, Kagawa S, et al. (2001) Gene expression profiling of clear cell renal cell carcinoma: gene identification and prognostic classification. Proc Natl Acad Sci U S A 98: 9754-9759.

67. Tanwar MK, Gilbert MR, Holland EC (2002) Gene expression microarray analysis reveals YKL-40 to be a potential serum marker for malignant character in human glioma. Cancer Res 62: 4364-4368.

68. Terris B, Blaveri E, Crnogorac-Jurcevic T, Jones M, Missiaglia E, et al. (2002) Characterization of gene expression profiles in intraductal papillary-mucinous tumors of the pancreas. Am J Pathol 160: 1745-1754.

69. Thieblemont C, Chettab K, Felman P, Callet-Bauchu E, Traverse-Glehen A, et al. (2002) Identification and validation of seven genes, as potential markers, for the differential diagnosis of small B cell lymphomas (small lymphocytic lymphoma, marginal zone B cell lymphoma and mantle cell lymphoma) by cDNA macroarrays analysis. Leukemia 16: 2326-2329.

70. van de Vijver MJ, He YD, van't Veer LJ, Dai H, Hart AA, et al. (2002) A gene-expression signature as a predictor of survival in breast cancer. N Engl J Med 347: 1999-2009.

71. van 't Veer LJ, Dai H, van de Vijver MJ, He YD, Hart AA, et al. (2002) Gene expression profiling predicts clinical outcome of breast cancer. Nature 415: 530-536.

72. Virtanen C, Ishikawa Y, Honjoh D, Kimura M, Shimane M, et al. (2002) Integrated classification of lung tumors and cell lines by expression profiling. Proc Natl Acad Sci U S A 99: 12357-12362.

73. Virtaneva K, Wright FA, Tanner SM, Yuan B, Lemon WJ, et al. (2001) Expression profiling reveals fundamental biological differences in acute myeloid leukemia with isolated trisomy 8 and normal cytogenetics. Proc Natl Acad Sci U S A 98: 1124-1129.

74. Wang E, Miller LD, Ohnmacht GA, Mocellin S, Perez-Diez A, et al. (2002) Prospective molecular profiling of melanoma metastases suggests classifiers of immune responsiveness. Cancer Res 62: 3581-3586.

75. Watson MA, Gutmann DH, Peterson K, Chicoine MR, Kleinschmidt-DeMasters BK, et al. (2002) Molecular characterization of human meningiomas by gene expression profiling using high-density oligonucleotide microarrays. Am J Pathol 161: 665-672.

76. Welsh JB, Sapinoso LM, Su AI, Kern SG, Wang-Rodriguez J, et al. (2001) Analysis of gene expression identifies candidate markers and pharmacological targets in prostate cancer. Cancer Res 61: 5974-5978.

77. Welsh JB, Zarrinkar PP, Sapinoso LM, Kern SG, Behling CA, et al. (2001) Analysis of gene expression profiles in normal and neoplastic ovarian tissue samples identifies candidate molecular markers of epithelial ovarian cancer. Proc Natl Acad Sci U S A 98: 1176-1181.

78. West M, Blanchette C, Dressman H, Huang E, Ishida S, et al. (2001) Predicting the clinical status of human breast cancer by using gene expression profiles. Proc Natl Acad Sci U S A 98: 11462-11467.

79. Wigle DA, Jurisica I, Radulovich N, Pintilie M, Rossant J, et al. (2002) Molecular profiling of non-small cell lung cancer and correlation with disease-free survival. Cancer Res 62: 3005-3008.

80. Yamanaka Y, Hamazaki Y, Sato Y, Ito K, Watanabe K, et al. (2002) Maturational sequence of neuroblastoma revealed by molecular analysis on cDNA microarrays. Int J Oncol 21: 803-807.

81. Yamazaki K, Sakamoto M, Ohta T, Kanai Y, Ohki M, et al. (2003) Overexpression of KIT in chromophobe renal cell carcinoma. Oncogene 22: 847-852.

82. Yanagawa R, Furukawa Y, Tsunoda T, Kitahara O, Kameyama M, et al. (2001) Genome-wide screening of genes showing altered expression in liver metastases of human colorectal cancers by cDNA microarray. Neoplasia 3: 395-401.

83. Ye QH, Qin LX, Forgues M, He P, Kim JW, et al. (2003) Predicting hepatitis B virus-positive metastatic hepatocellular carcinomas using gene expression profiling and supervised machine learning. Nat Med 9: 416-423.

84. Yeoh EJ, Ross ME, Shurtleff SA, Williams WK, Patel D, et al. (2002) Classification, subtype discovery, and prediction of outcome in pediatric acute lymphoblastic leukemia by gene expression profiling. Cancer Cell 1: 133-143.

85. Zhan F, Hardin J, Kordsmeier B, Bumm K, Zheng M, et al. (2002) Global gene expression profiling of multiple myeloma, monoclonal gammopathy of undetermined significance, and normal bone marrow plasma cells. Blood 99: 1745-1757.
