# Supplementary material for: Sharing Detailed Research Data Is Associated with Increased Citation Rate
Source: PLoS One. 2007 Mar 21;2(3):e308. doi: 10.1371/journal.pone.0000308 (PMC1817752; doi:10.1371/journal.pone.0000308)
Supplement: Text S2 — Locations of Publicly Available Data for the Cohort (0.05 MB DOC) [file pone.0000308.s002.doc]

#### Sharing Detailed Research Data is

#### Associated with Increased Citation Rate

**Locations of Public Microarray Data**

Adeyinka A, 2002, Clin Cancer Res, V8, P3788

Ahr A, 2002, Lancet, V359, P131

Alizadeh AA, 2000, Nature, V403, P503

http://llmpp.nih.gov/lymphoma/analysis.shtml

http://smd.stanford.edu/cgi-bin//publication/viewPublication.pl?pub_no=79

http://www.ncbi.nlm.nih.gov/entrez/query.fcgi?CMD=search&DB=gds

http://www.ebi.ac.uk/aerep/dataselection?expid=500327225

Bayani J, 2002, Cancer Res, V62, P3466

http://www.utoronto.ca/cancyto/OVCA2001CR/

Beer DG, 2002, Nat Med, V8, P816

http://dot.ped.med.umich.edu:2000/ourimage/pub/Lung/index.html

Belbin TJ, 2002, Cancer Res, V62, P1184

Bertucci F, 2002, Hum Mol Genet, V11, P863

Bhattacharjee A, 2001, P Natl Acad Sci USA, V98, P13790

http://research.dfci.harvard.edu/meyersonlab/lungca/

BirkenkampDemtroder K, 2002, Cancer Res, V62, P4352

Bittner M, 2000, Nature, V406, P536

http://www.nature.com/nature/journal/v406/n6795/suppinfo/406536a0.html

http://www.ncbi.nlm.nih.gov/entrez/query.fcgi?db=pubmed&cmd=Display&dopt=pubmed_gds&from_uid=10952317

Bohen SP, 2003, P Natl Acad Sci USA, V100, P1926

http://genome-www.stanford.edu/rituximab/

Cohen N, 2001, Cancer Genet Cytogen, V128, P114

Delpuech O, 2002, Oncogene, V21, P2926

Devilard E, 2002, Oncogene, V21, P3095

Dhanasekaran SM, 2001, Nature, V412, P822

http://www.pathology.med.umich.edu/chinnaiyan/Nature/Nature.htm

Dyrskjot L, 2003, Nat Genet, V33, P90

http://www.ncbi.nlm.nih.gov/geo/query/acc.cgi?acc=GSE89

http://www.nature.com/ng/journal/v33/n1/suppinfo/ng1061_S1.html

ElNaggar AK, 2002, Oncogene, V21, P8206

FathallahShaykh HM, 2002, Oncogene, V21, P7164

Fritz B, 2002, Cancer Res, V62, P2993

Fuller GN, 1999, Cancer Res, V59, P4228

Fuller GN, 2002, Brain Pathol, V12, P108

Garber ME, 2001, P Natl Acad Sci USA, V98, P13784

http://genome-www.stanford.edu/lung_cancer/adeno/data.shtml

http://www.pnas.org/cgi/content/full/241500798/DC1?maxtoshow=&HITS=10&hits=10&RESULTFORMAT=1&author1=Garber%2C+ME&andorexacttitle=and&andorexacttitleabs=and&andorexactfulltext=and&searchid=1122046521379_4438&stored_search=&FIRSTINDEX=0&sortspec=relevance&fdate=7/1/1995&journalcode=pnas

http://www.ebi.ac.uk/aerep/result?queryFor=Experiment&eAccession=E-SMDB-810

Golub TR, 1999, Science, V286, P531

http://www-genome.wi.mit.edu/cgi-bin/cancer/datasets.cgi

Gruvberger S, 2001, Cancer Res, V61, P5979

http://research.nhgri.nih.gov/microarray/ER_data.txt

Gutmann DH, 2002, Cancer Res, V62, P2085

http://bioinformatics.wustl.edu/mgacore/servlet/Publications#reference3

Hedenfalk I, 2001, New Engl J Med, V344, P539

http://research.nhgri.nih.gov/microarray/NEJM_Supplement/

http://www.nejm.org

Hippo Y, 2002, Cancer Res, V62, P233

http://cancerres.aacrjournals.org/cgi/content/full/62/1/233/DC1

Hofmann WK, 2002, Lancet, V359, P481

Huang HT, 2000, Cancer Res, V60, P6868

http://thinker.med.ohio-state.edu

Iizuka N, 2002, Cancer Res, V62, P3939

Iizuka N, 2003, Lancet, V361, P923

http://surgery2.med.yamaguchi-u.ac.jp/research/DNAchip/hcc-recurrence/index.html

Jazaeri AA, 2002, J Natl Cancer I, V94, P990

http://jncicancerspectrum.oupjournals.org/cgi/content/full/jnci;94/13/990/DC1

Jazaeri AA, 2003, Mol Carcinogen, V36, P53

Kihara C, 2001, Cancer Res, V61, P6474

Klein U, 2001, J Exp Med, V194, P1625

http://www.jem.org/cgi/content/full/194/11/1625/F2/DC1

LaTulippe E, 2002, Cancer Res, V62, P4499 http://gedp.nci.nih.gov/dc/servlet/manager

http://cancerres.aacrjournals.org/cgi/content/full/62/15/4499/DC1

Lee S, 2002, Cancer Lett, V184, P197

Luo JH, 2002, Mol Carcinogen, V33, P25

MacDonald TJ, 2001, Nat Genet, V29, P143

http://microarray.cnmcresearch.org/cancer_human.htm

http://www.ncbi.nlm.nih.gov/projects/geo/gds/gds_browse.cgi?gds=232

Magee JA, 2001, Cancer Res, V61, P5692

http://bioinformatics.wustl.edu/mgacore/servlet/Publications

Mendez E, 2002, Cancer, V95, P1482

http://www.fhcrc.org/science/labs/chen/

Miura K, 2002, Cancer Res, V62, P3244

Moos PJ, 2002, Clin Cancer Res, V8, P3118

Moran CJ, 2002, Clin Cancer Res, V8, P3803

Mukasa A, 2002, Oncogene, V21, P3961

Notterman DA, 2001, Cancer Res, V61, P3124

http://microarray.princeton.edu/oncology/database.html

Ohmine K, 2001, Oncogene, V20, P8249

Okabe H, 2001, Cancer Res, V61, P2129

Perou CM, 1999, P Natl Acad Sci USA, V96, P9212

http://genome-www.stanford.edu/molecularportraits/

http://www.pnas.org/cgi/content/full/96/16/9212/DC1/2

http://smd.stanford.edu/cgi-bin//publication/viewPublication.pl?pub_no=6

Pomeroy SL, 2002, Nature, V415, P436

http://www-genome.wi.mit.edu/cgi-bin/cancer/publications/pub_paper.cgi?mode=view&paper_id=52

http://www.nature.com/nature/journal/v415/n6870/abs/415436a_fs.html

Rickman DS, 2001, Cancer Res, V61, P6885

http://gedp.nci.nih.gov/dcfiles/datafiles/

Rodriguez S, 2003, Oncogene, V22, P1880

http://myprofile.cos.com/chenabcd45

Rosenwald A, 2001, J Exp Med, V194, P1639

http://llmpp.nih.gov/MCL/

http://smd.stanford.edu/cgi-bin//publication/viewPublication.pl?pub_no=104

Rosenwald A, 2002, New Engl J Med, V346, P1937

http://llmpp.nih.gov/DLBCL/

http://online6.hsls.pitt.edu:2305/cgi/content/full/346/25/1937/DC1

Schoch C, 2002, P Natl Acad Sci USA, V99, P10008

http://www.pnas.org/cgi/content/full/142103599/DC1

Shipp MA, 2002, Nat Med, V8, P68

http://www-genome.wi.mit.edu/mpr/lymphoma/

Shirota Y, 2001, Hepatology, V33, P832

Shridhar V, 2001, Cancer Res, V61, P5895

Signoretti S, 2002, J Clin Invest, V110, P633

Singh D, 2002, Cancer Cell, V1, P203

http://www-genome.wi.mit.edu/MPR/prostate

http://www.cancercell.org/cgi/content/full/1/2/203/

Skotheim RI, 2002, Cancer Res, V62, P2359

Smith MW, 2003, Cancer Res, V63, P859

http://cancerres.aacrjournals.org/cgi/data/63/4/859/DC1/1

Sorlie T, 2001, P Natl Acad Sci USA, V98, P10869

http://genome-www5.stanford.edu/cgi-bin/SMD/publication/viewPublication.pl?pub_no=95

Sotiriou C, 2002, Breast Cancer Res, V4

Stratowa C, 2001, Int J Cancer, V91, P474

Takahashi M, 2001, P Natl Acad Sci USA, V98, P9754

http://www.pnas.org/cgi/content/full/171209998/DC1?maxtoshow=&HITS=10&hits=10&RESULTFORMAT=1&author1=takahashi&title=gene+expression+profiling&andorexacttitle=and&andorexacttitleabs=and&andorexactfulltext=and&searchid=1122170397314_2926&stored_search=&FIRSTINDEX=0&sortspec=relevance&fdate=7/1/1995&journalcode=pnas

Tanwar MK, 2002, Cancer Res, V62, P4364

Terris B, 2002, Am J Pathol, V160, P1745

Thieblemont C, 2002, Leukemia, V16, P2326

vandeVijver MJ, 2002, New Engl J Med, V347, P1999

http://www.rii.com/publications/2002/nejm.html

vantVeer LJ, 2002, Nature, V415, P530

http://www.rii.com/publications/2002/vantveer.htm

http://www.nature.com/nature/journal/v415/n6871/abs/415530a_fs.html

Virtanen C, 2002, P Natl Acad Sci USA, V99, P12357

http://www.pnas.org/cgi/content/full/192240599/DC1?maxtoshow=&HITS=10&hits=10&RESULTFORMAT=&fulltext=Virtanen&searchid=1122290569436_2052&stored_search=&FIRSTINDEX=0&journalcode=pnas

Virtaneva K, 2001, P Natl Acad Sci USA, V98, P1124

http://thinker.med.ohio-state.edu/aml/index.html

Wang E, 2002, Cancer Res, V62, P3581

Watson MA, 2002, Am J Pathol, V161, P665

http://bioinformatics.wustl.edu/mgacore/servlet/Publications#reference1

Welsh JB, 2001, Cancer Res, V61, P5974

http://www.gnf.org/cancer/prostate

Welsh JB, 2001, P Natl Acad Sci USA, V98, P1176

http://www.gnf.org/cancer/ovary/

http://www.pnas.org/cgi/search?sendit=Search&pubdate_year=&volume=&firstpage=&DOI=&author1=welsh&author2=&title=epithelial+ovarian&andorexacttitle=and&titleabstract=&andorexacttitleabs=and&fulltext=&andorexactfulltext=and&fmonth=Jul&fyear=1995&tmonth=Jul&tyear=2005&fdatedef=1+January+1915&tdatedef=26+July+2005&tocsectionid=all&RESULTFORMAT=1&hits=10&hitsbrief=25&sortspec=relevance&sortspecbrief=relevance

West M, 2001, P Natl Acad Sci USA, V98, P11462

http://www.pnas.org/cgi/content/full/201162998/DC1?maxtoshow=&HITS=10&hits=10&RESULTFORMAT=1&author1=West&andorexacttitle=and&andorexacttitleabs=and&andorexactfulltext=and&searchid=1122412928335_7171&stored_search=&FIRSTINDEX=0&sortspec=relevance&fdate=7/1/1995&journalcode=pnas

http://data.cgt.duke.edu/

Wigle DA, 2002, Cancer Res, V62, P3005

http://www.cs.utoronto.ca/~juris/publicationsData.html

http://www.fhcrc.org/science/labs/chen/

Yamanaka Y, 2002, Int J Oncol, V21, P803

Yamazaki K, 2003, Oncogene, V22, P847

Yanagawa R, 2001, Neoplasia, V3, P395

Ye QH, 2003, Nat Med, V9, P416

http://www.ncbi.nlm.nih.gov/geo/query/acc.cgi?acc=GSE364

http://www.nature.com/nm/journal/v9/n4/suppinfo/nm843_S1.html

Yeoh EJ, 2002, Cancer Cell, V1, P133

http://www.stjuderesearch.org/data/ALL1

Zhan FH, 2002, Blood, V99, P1745

http://lambertlab.uams.edu/software/
